# Transcriptome profiling of zebrafish optic fissure fusion

**DOI:** 10.1038/s41598-018-38379-5

**Published:** 2019-02-07

**Authors:** R. Richardson, N. Owen, M. Toms, Rodrigo M. Young, D. Tracey-White, M. Moosajee

**Affiliations:** 1 Development, Ageing and Disease, UCL Institute of Ophthalmology, London, UK; 2 Department of Genetics, Moorfields Eye Hospital NHS Foundation Trust, London, UK; 3 Department of Ophthalmology, Great Ormond Street Hospital for Children NHS Foundation Trust, London, UK

## Abstract

Incomplete fusion of the optic fissure leads to ocular coloboma, a congenital eye defect that affects up to 7.5 per 10,000 births and accounts for up to 10 percent of childhood blindness. The molecular and cellular mechanisms that facilitate optic fissure fusion remain elusive. We have profiled global gene expression during optic fissure morphogenesis by transcriptome analysis of tissue dissected from the margins of the zebrafish optic fissure and the opposing dorsal retina before (32 hours post fertilisation, hpf), during (48 hpf) and after (56 hpf) optic fissure fusion. Differential expression analysis between optic fissure and dorsal retinal tissue resulted in the detection of several known and novel developmental genes. The expression of selected genes was validated by qRT-PCR analysis and localisation investigated using *in situ* hybridisation. We discuss significantly overrepresented functional ontology categories in the context of optic fissure morphogenesis and highlight interesting transcripts from hierarchical clustering for subsequent analysis. We have identified netrin1a (*ntn1a)* as highly differentially expressed across optic fissure fusion, with a resultant ocular coloboma phenotype following morpholino antisense translation-blocking knockdown and downstream disruption of *atoh7* expression. To support the identification of candidate genes in human studies, we have generated an online open-access resource for fast and simple quantitative querying of the gene expression data. Our study represents the first comprehensive analysis of the zebrafish optic fissure transcriptome and provides a valuable resource to facilitate our understanding of the complex aetiology of ocular coloboma.

## Introduction

Epithelial fusion events during embryogenesis are crucial for the correct formation and function of multiple organs and tissues. Fusion requires precise spatiotemporal molecular control of cell migration, proliferation and programmed cell death^1^. During ocular development, the neuroectodermal layers of the optic vesicle invaginate to form a bi-layered optic cup. The invagination process leads to the formation of a transient opening along the ventral aspect of the retina and optic stalk, called the optic fissure, through which the hyaloid artery and vein enter and supply the developing eye^2–4^. Fusion of the optic fissure, which normally occurs during weeks 5 to 7 of human gestation, involves apposition of the epithelial margins around the vasculature, spatial specification along the proximal-distal axis of the fissure and basement membrane breakdown, resulting in the formation of a continuous epithelial layer^5–7^.

Incomplete fusion of the optic fissure leads to the congenital eye defect ocular coloboma, located in the inferonasal quadrant of the eye. It can involve one or multiple ocular tissues spanning the iris, zonules and ciliary body, retina, choroid and optic nerve^8,9^. Ocular coloboma has a prevalence of up to 7.5 per 10,000 births and accounts for approximately 10% of childhood blindness worldwide^10,11^. Ocular coloboma can present in isolation, as part of a clinical spectrum with microphthalmia and anophthalmia (mixed), associated with other ocular disorders (complex) or with other systemic features (syndromic)^12^. To date, around 100 genes have been associated with non-syndromic and syndromic ocular coloboma, microphthalmia and anophthalmia, showing extensive genetic heterogeneity and complexity^12,13^.

Zebrafish eye development displays molecular complexity and stringent spatiotemporal regulation which is similar to that seen in humans^14,15^. Our objective was to analyse transcriptome changes in the zebrafish optic fissure before (32 hours post fertilisation, hpf), during (48 hpf) and after fissure fusion (56 hpf). Thus, we analysed global gene expression in tissue dissected from the margins of the optic fissure and opposing dorsal retina tissue. In zebrafish, a superior ocular sulcus also extends across the dorsal retina, separating the nasal and temporal retinal lobes, however this sulcus is present transiently, closing by 26 hpf^16^.

We discuss biological themes inferred from gene ontology (GO) overrepresentation analysis in the context of optic fissure morphogenesis. Hierarchical clustering facilitated the detection of homogeneous co-expressed gene subgroups, including known and predicted unknown genes underlying fusion of the optic fissure, thus expanding the coloboma target gene repertoire for screening, diagnostics and further functional studies. We further characterised candidates *fam132a* and *ntn1a* by gene silencing via morpholino antisense translation-blocking knockdown. Finally, we have used our dataset to generate an open access resource for fast and simple quantitative querying of the RNA-seq gene expression data (bit.ly/ZfOptic2018).

## Results

### Transcriptome sequencing and mapping

**Figure 1:**
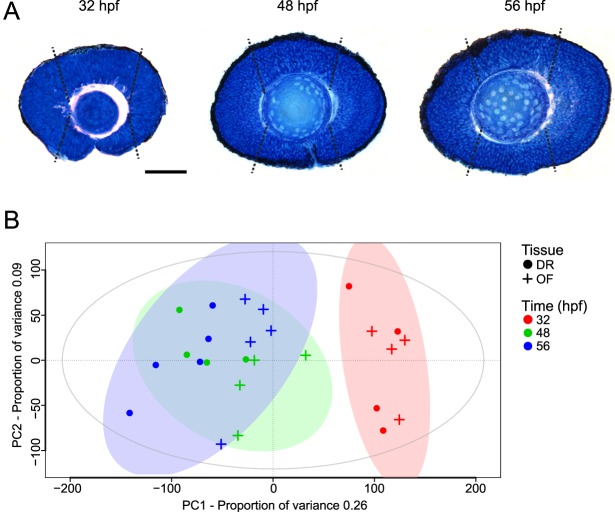
Optic fissure (OF) and dorsal retina (DR) tissue collection and principle component analysis (PCA). (**A**) Sagittal section through the zebrafish eye at the level of the lens at 32, 48 and 56 hpf, representative of before, during and after optic fissure fusion. White dotted lines indicate the tissue (inferior is optic fissure and superior is dorsal retina) harvested for RNA-seq transcriptome analysis. Scale bar 50 *µ*m. (**B**) PCA of RNA-seq transcriptome data expression patterns using regularized-logarithm transformed count data in DESeq2. Time (hours post fertilization, hpf) is represented by colour and origin of tissue by shape.

To investigate the mechanisms that underpin optic fissure fusion, we carried out RNA transcriptome analysis using high quality mRNA extracted from wild-type, AB-strain zebrafish optic fissure (OF) and opposing dorsal retina (DR) tissue (Fig. 1A). Time points 32 hpf, 48 hpf and 56 hpf were chosen as representative of before, during and after optic fissure fusion (Fig. 1A). Tissue was dissected from five biological replicates at each time point for sufficient statistical power. Four of the thirty samples failed library read duplication quality control and were removed from subsequent analysis (paired OF and DR samples at 32 hpf and 48 hpf). High quality reads were mapped to the reference zebrafish genome GRCz10. A summary of read alignment metrics for each sample is shown in Table S1. Principle component analysis (PCA) clustered the 48 hpf and 56 hpf OF and DR samples, distinct from 32 hpf OF and DR samples emphasising less biological variance between samples at 48 hpf and 56 hpf, whilst 32 hpf demonstrated a more disparate transcriptome signature (Fig. 1B).

### OF versus DR pairwise tissue analysis

To identify differentially expressed gene (DEG) events within the data we carried out pairwise condition testing. All possible discrete combinations of tissue type and time point conditions were tested, identifying DEGs with a fold change greater than two and adjusted *p*-value below 0.01. The filtered data output for each test are listed in Table S2. We prioritised pairwise OF versus reference DR tissue tests at 32 hpf, 48 hpf and 56 hpf to identify biologically important DEG events for proper optic fissure morphogenesis. DEGs were reported in relation to the expression in reference DR tissue at each time point. At 32 hpf, we identified 181 DEGs in OF tissue; 146 DEGs were upregulated and 35 downregulated in OF tissue relative to the DR. At 48 hpf, 79 DEGs were identified; 63 DEGs were upregulated and 16 downregulated in OF tissue. Finally, at 56 hpf, we identified 62 DEGs; 44 DEGs were upregulated and 18 downregulated in OF tissue (Table S2).

**Figure 2:**
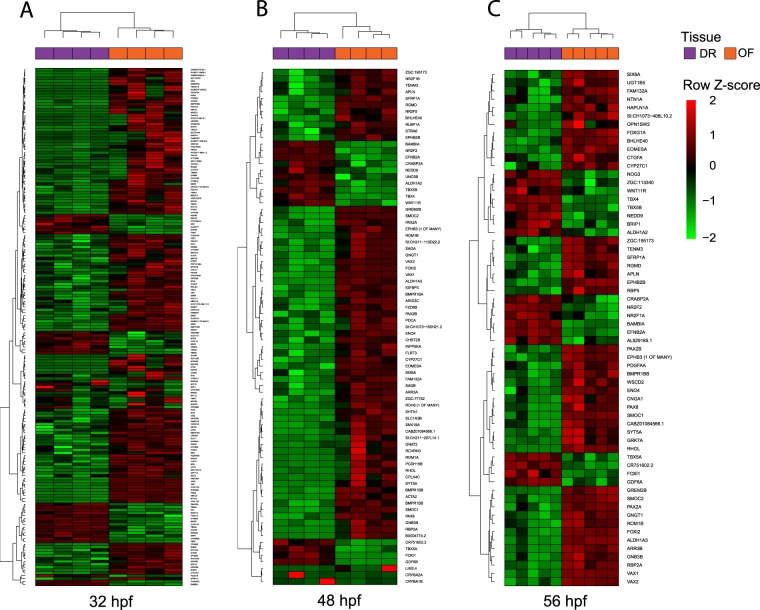
Hierarchical clustered heatmaps illustrating significant differentially expressed gene (DEG) profiles between optic fissure (OF) and opposing dorsal retina (DR) tissue. DEG events between OF versus DR pairwise tests at (**A**) 32 hpf, (**B**) 48 hpf and (**C**) 56 hpf. Rows indicate DEG genes; columns represent individual tissue samples from the time points.

We performed unbiased hierarchical cluster analysis on significant DEG events at each time point to identify co-regulated or functionally related DEGs. The data were visualised using heat maps (Fig. 2A–C). Individual clusters were assigned to general groups following visual examination of the expression patterns. Gene ontology enrichment analysis was performed on clusters with positive fold change increase. At 32 hpf clusters of genes with higher expression in OF versus DR showed an enrichment for ‘regulation of neurogenesis’ (GO:0050767) and ‘embryonic morphogenesis’ (GO:0048598) [*gna2*, *nr2f5*, *vax2*, *rom1a*, *arr3a*, *rhoI*]. At 48 hpf, we identified ontologies including: ‘visual perception’ (GO:0007601), ‘embryonic camera-type eye morphogenesis’ (GO:0048596) [*pax2a*, *vax1*, *vax2* and *sfrp1a*], as well as ‘closure of optic fissure’ (GO:0061386) with *vax1*, *vax2* and *sfrp1a* as the most significant group. Clustering genes higher expressed in OF versus DR at 56 hpf identified ‘closure of optic fissure’ (GO:0061386).

### Overrepresentation analysis of DEG events during optic fissure morphogenesis

**Figure 3:**
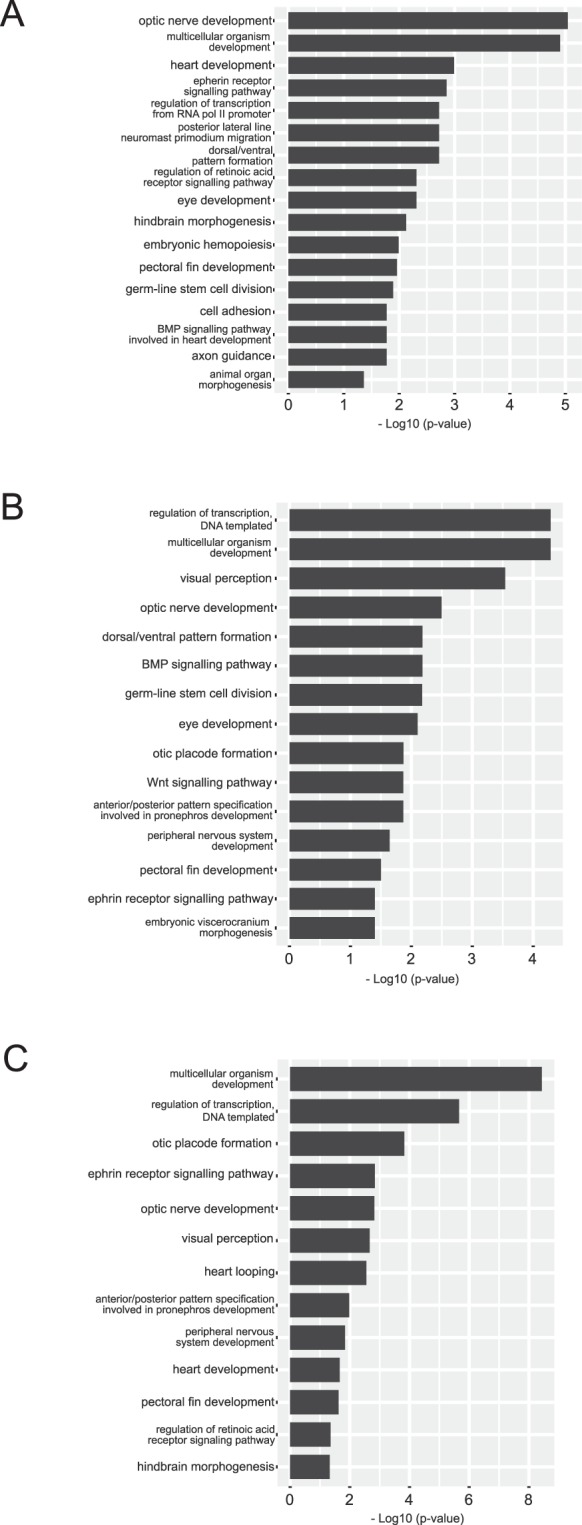
Gene ontology overrepresentation analysis of significant differentially expressed gene (DEG) events between optic fissure (OF) and opposing dorsal retina (DR) tissue. Assigned gene ontology (GO) terms were used to classify functions of DEGs based on biological process at (**A**) 32 hpf, (**B**) 48 hpf and (**C**) 56 hpf. The plots show significantly enriched GO terms at each time point. Detailed information can be found in Table S3.

To classify gene sets functionally, we conducted overrepresentation analysis of statistically significant DEG events between OF and DR tissue at each time point and subset ‘Biological process’ gene ontology (GO) terms with the aim to delineate processes governing optic fissure fusion. At 32 hpf, 34 significantly overrepresented GO terms derived from the ‘Biological Process’ category were identified, 22 terms at 48 hpf and 24 terms at 56 hpf. A detailed summary of significantly overrepresented GO terms at each time point is provided in Table S3. Significantly overrepresented GO terms, after multiple testing adjustment, for each time point tissue comparison are shown in Fig. 3.

#### Ocular development and function

At each time point investigated, DEG events were significantly overrepresented for GO terms linked to ocular development, including ‘eye development’ (GO:0001654), ‘retina morphogenesis in camera-type eye’ (GO:0060042) and ‘optic nerve development’ (GO:0021554). Importantly, at 48 hpf and 56 hpf DEG events were significantly overrepresented for the GO term, ‘optic fissure fusion’ (GO:0061386); specifically, we observed upregulation of *vax2* (LFC [log2FoldChange] 5.60, *p* < 0.001 at 48 hpf; LFC 3.98, *p* < 0.001 at 56 hpf) and *sfrp1a* (LFC 2.19, *p* < 0.001 at 48 hpf; LFC 2.29, *p* < 0.001 at 56 hpf) in OF relative to DR tissue. Both *vax2* and *sfrp1a* genes have been implicated in dorsoventral patterning of the developing eye and their loss of function leads to ocular coloboma^17,18^.

Throughout 32–56 hpf, DEG events were also significantly overrepresented for GO terms linked to ocular function, such as, ‘phototransduction’ (GO:0007602), ‘detection of light stimulus involved in visual perception’ (GO:0050908), ‘visual perception’ (GO:0007601) and ‘photoreceptor activity’ (GO:0009881).

#### Retinal patterning and signal transduction pathways

Patterning of the developing eye is critically important for proper axonal pathfinding and morphogenetic events, such as optic fissure fusion^19^. In our pairwise tissue DEG data GO terms, ‘dorsal/ventral pattern formation’ (GO:0009953), ‘anterior/posterior pattern specification’ (GO:0009952) and ‘determination of dorsal identity’ (GO:0048263) were overrepresented.

Wnt, Bmp4 and RA signalling pathways in the dorsal retina play significant roles in establishing dorsoventral retinal boundaries and related morphogenetic movements^20^. Accordingly, GO terms, ‘regulation of RA receptor signalling pathway’ (GO:0048385), ‘BMP signalling pathway’ (GO:0030509) and ‘negative regulation of BMP signalling pathway’ (GO:0030514) were overrepresented between 32–56 hpf alongside terms, ‘Wnt signalling pathway’ (GO:0016055) and ‘Positive regulation of Wnt signalling pathway (GO:0090263). For example, at 32 hpf, we observed downregulation of *bmp4* (LFC −1.64, *p* < 0.001) in OF tissue as well as down-regulation of putative BMP target, *tbx5a* (LFC −9.29, *p* < 0.001) and BMP and activin membrane bound inhibitor, *bambia* (LFC −3.38, *p* < 0.001) in OF relative to DR. Additionally, we observed downregulation of *wnt11r* at 48–56 hpf in OF (LFC −2.21 at 48 hpf, LFC −1.84 at 56 hpf, *p* < 0.01). Importantly, optic fissure fusion defects have been linked to aberrant dorsal-ventral neuro-retinal patterning and the spatiotemporal interaction of signalling pathways in normal optic vesicle development has been described^3,21^.

#### Periocular mesenchyme (POM) development and function

During optic fissure morphogenesis, POM cells, which arise from both the head mesoderm and neural crest, migrate into the optic cup through the optic fissure to form the retinal vasculature^22,23^. POM-derived endothelial cells that give rise to the hyaloid vasculature contribute to basement membrane breakdown, extending processes to contact the cells of the optic fissure margins to facilitate fusion^5,24^. At 32 hpf, the GO category ‘mesodermal cell migration’ (GO:0008078), was found to be significantly over-represented; specifically, we observed upregulation of genes encoding Apelin (*apln*) (LFC 2.69, *p* < 0.001) and its cognate receptor *aplnrb* (LFC 1.43, *p* < 0.01), in OF tissue at 32 hpf. At 48 hpf and 56 hpf, these genes were no longer significantly differentially expressed in OF samples, in keeping with the observed presence of mesodermal-derived POM cells within the fissure up until, but not after optic fissure fusion^24^. Importantly. mutations in genes controlling POM development or function have been shown to disrupt optic fissure fusion^25^.

#### Vascular development

The hyaloid vasculature and/or POM-derived endothelial cells giving rise to the hyaloid vasculature enter the optic cup through the optic fissure and contribute to basement membrane breakdown and fissure fusion^5^. DEG events were significantly overrepresented for GO terms ‘embryonic hemopoiesis’ (GO:0035162), ‘angiogenesis’ (GO:0001525), ‘negative regulation of angiogenesis’ (GO:0016525), ‘vasculogenesis’ (GO:0001570), and ‘vasculature development’ (GO:0001944), at 32 hpf. Specifically, at 32 hpf we observed upregulated expression of vasculo- and angio-genesis regulators including; *ntn1a* (LFC 5.52, *p* < 0.001), *klf2a* (LFC 2.81, *p* < 0.001), *adgrb3* (LFC 5.19, *p* < 0.01), *tbx20* (LFC 4.32, *p* < 0.001) and *slc4a1a* (LFC 4.63, *p* < 0.001) in OF tissue, alongside DR DEGs *unc5b* (LFC -3.14, *p* < 0.001) and *efnb2a* (LFC -2.93, *p* < 0.001).

By 48 hpf the margins of the optic fissure come together, enclosing the hyaloid artery and vein. At 48 hpf the GO term, ‘venous blood vessel development’ (GO:0060841) was significantly overrepresented, with vascular specification markers *nr2f2* (LFC -1.63, *p* < 0.001) and *nr2f1b* (LFC 1.22, *p* < 0.01) showing differential expression between the OF and DR. Functional redundancy of genes involved in hematopoiesis and vascular development can lead to failed optic fissure fusion^26^.

#### Cell-cell interactions

After mesenchymal cell migration, the margins of the optic fissure align and fuse forming a continuous optic cup. This process requires complex cell morphological changes and rearrangement at the optic fissure margin, largely mediated by the structural remodelling of cell-cell adhesions^27,28^. For example, cell adhesion molecules regulate homotypic interactions between projecting axons. In this context, between 30–36 hpf the first (RGC) axons exit and navigate along the optic stalk^29^. At 32 hpf DEG events were overrepresented for GO terms, ‘cell adhesion’ (GO:0007155) and ‘homophilic cell adhesion via plasma membrane adhesion molecules’ (GO:0007156). Specifically, we observed upregulation of cadherin superfamily genes *cdh4* (LFC 1.93, *p* < 0.001), *pcdh8* (LFC 2.72, *p* < 0.001), *pcdh11* (LFC 4.19, *p* < 0.001) and *pcdh12* (LFC 3.61, *p* < 0.01) in OF tissue.

### Validation and visualisation of mRNA expression

**Figure 4:**
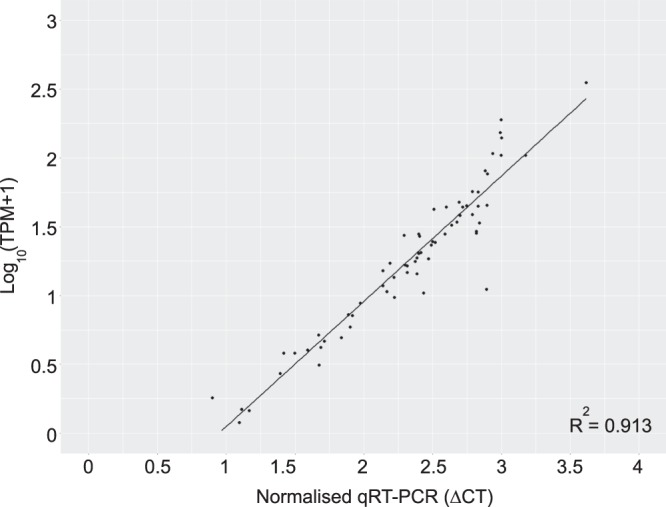
Gene expression correlation between qRT-PCR and RNA-seq trancriptome data. The plot shows gene expression abundance determined by qRT-PCR and RNA-seq transcriptome analysis for 11 selected genes. TPMs were calculated using the online resource generated for this manuscript (bit.ly/ZfOptic2018). The Pearson correlation coefficient (R^2^) and linear regression line are indicated (*p* < 0.001).

We validated the expression of 11 selected genes using real-time quantitative RT-PCR (qRT-PCR) analysis. Genes *g6pd*, *znf644a*, *tbx3a*, *efnb2a*, *nxn*, *dacha*, *smad6a*, *msxc*, *bambia*, *src* and *arl3l1* were selected due to their distribution across varied GO hierarchy (Table S3). To evaluate concordance in gene expression intensities between RNA-seq and qRT-PCR data, we applied Spearman’s correlation coefficient. The Spearman correlation coefficient was 0.913 (*p* < 0.001) for selected genes (Fig. 4), indicating the high reliability and accuracy of our RNA-seq transcriptome analysis to quantify gene expression during zebrafish optic fissure morphogenesis.

**Figure 5:**
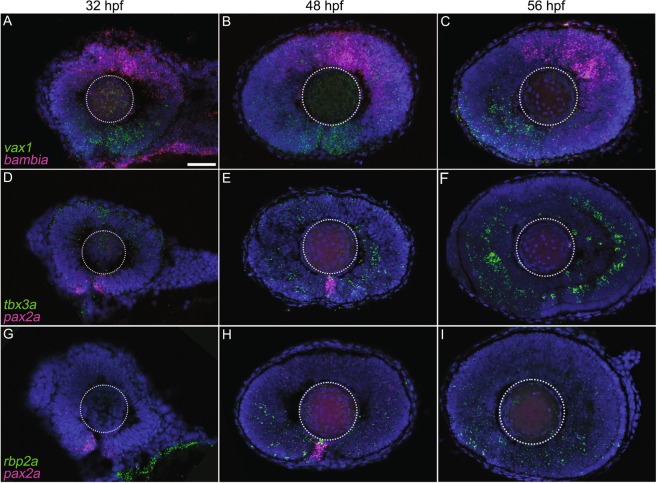
RNAscope *in situ* hybridisation for visualisation of selected differentially expressed gene (DEG) events from RNA-seq transcriptome analysis. Representative images of dual *in situ* hybridisation in the wild-type zebrafish retina using mRNA probes to (**A**–**C**) *vax1* and *bambia*, (**D**–**F**) *tbx3a* and *pax2a*, and (**G**–**I**) *rbp2a* and *pax2a* at 32, 48 and 56 hpf. Nuclei were labelled with 4′,6-diamidino-2-phenylindole (DAPI). White dotted lines indicate the location of the lens. Scale bar 50 *µ*m.

RNAscope *in situ* hybridisation (ISH) permitted the visualisation of mRNA transcripts providing spatial and morphological context to the expression data (Fig. 5). Transcripts selected were predicted to show distinct spatiotemporal expression patterns across the dorsal-ventral axis of the developing retina using the transcriptome analysis. Positive signal for *pax2a* mRNA was restricted to the margins of the optic fissure during fusion, with little/no expression once fusion was complete (Fig. 5D–I). Expression of *vax1* mRNA was confined to the developing ventral retina throughout optic fissure fusion (Fig. 5A–C). Expression of *rbp2a* was observed in the retina at 48 and 56 hpf, predominantly in the ventral region and with increasing expression over time (Fig. 5G–I). We also analysed the expression of BMP-antagonists *bambia* and *tbx3a*. Consistent with previous reports, expression of *bambia* was restricted to the dorsal developing retina^30^ (Fig. 5A–C). Expression of *tbx3a* was observed in the dorsal retina throughout 32–56 hpf and in the ventral retina from 48–56 hpf, with highest expression in the ventral retina (Fig. 5D–F).

### Global analysis of differentially expressed genes over time

To better define the dynamic changes in global gene expression levels between 32–56 hpf and to isolate high priority candidates that are biologically relevant during optic fissure fusion we performed a time series DEG analysis using the DESeq2 likelihood-ratio test (LRT). LRT aims to test whether a gene’s expression alters in a condition specific manner between OF and DR tissue over time. In analysing count data, DESeq2 estimates dispersion of each transcript’s expression by considering the dispersion of genes expressed at similar levels. Using this method, we identified 38 significant DEG events between OF and DR tissue over time (Table S5). Highly significant (p < 0.001) DEG events between OF and DR over time included T-box genes involved in heart development (*tbx2a/3a*), regulation of glucose (*fam132a*), membrane component (*chst2b*), regulation of vascular permeability (*src*), transmembrane transport (*slc6a17*), metabolic process (*pah*), retinoic acid regulation (*nr2f5)* and axon guidance (*arl3l1*, *ntn1a*, *tnc*).

**Figure 6:**
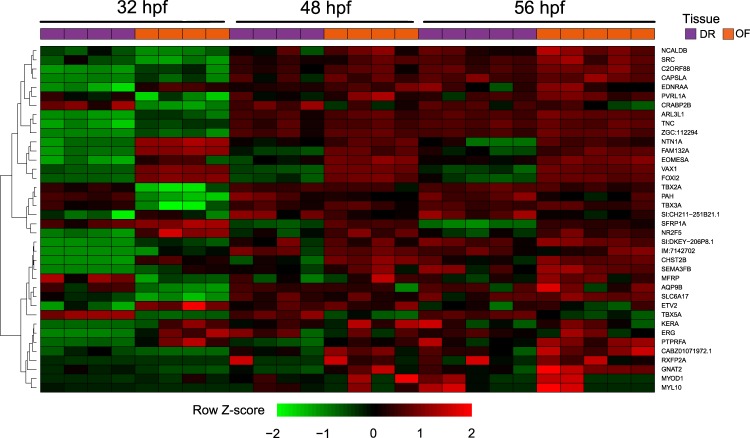
Global analysis of differentially expressed gene (DEG) events over time. The heatmap shows DEG events between optic fissure (OF) and dorsal retina (DR) tissue over time after being subjected to hierarchical cluster analysis.

To identify co-expression patterns between DEG events, we applied unbiased hierarchical cluster analysis to the data sets and visualised the data using heat maps (Fig. 6). Tissue-type/stage hierarchical clustering and PCA analysis (Fig. 1B) supported similar interpretations; 32 hpf OF and DR samples formed distinct clusters from 48–56 hpf OF and DR samples. Additionally, OF samples at 48 hpf and 56 hpf clustered together, and DR samples at 48 hpf and 56 hpf clustered together.

Of note, *ntn1a*, *fam132a* and *eomesa* clustered together with coloboma-causing *vax1*^31^ and forkhead box gene *foxi2*, which has a role in ocular size, localised in the ventral retina^32^. This ventral distribution of *foxi2* is significantly altered in zebrafish lacking gdf6a which results in a spectrum of microphthalmia, anophthalmia and coloboma (MAC) phenotypes. This gene set demonstrated upregulated expression in OF relative to DR tissue at 32 hpf, which was still evident but less pronounced by 48–56 hpf (Fig. 6).

### Functional investigation of highly significant DEGs identified using LRT analysis highlights *ntn1a* as a novel mediator of optic fissure fusion

**Figure 7:**
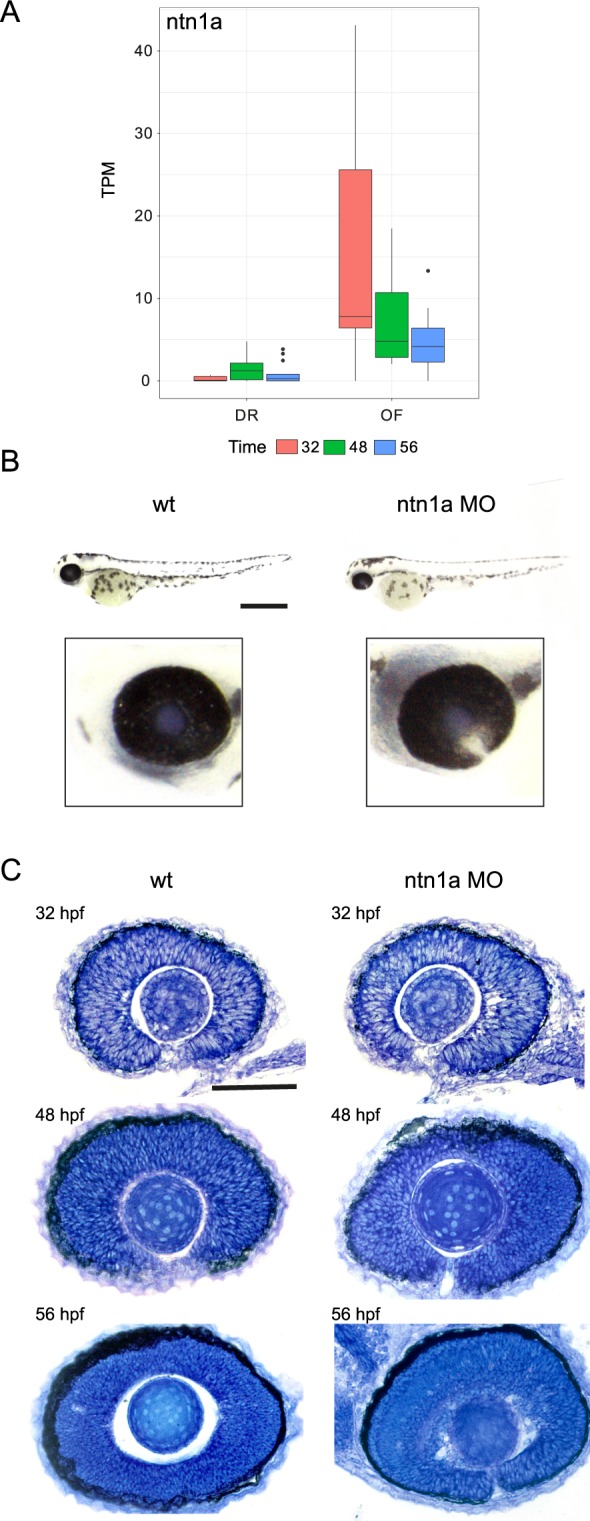
Failure of optic fissure fusion in *ntn1a* morphant zebrafish embryos. Expression levels (TPM, transcripts per million) of *ntn1a* candidate DEG showing temporal expression at 32,48 and 56 hours post fertilization (hpf) (**A**). (**B**) Wholemount and ocular morphology of 56 hpf wildtype (wt) and ntn1a morphant embryos, showing loss of ntn1a results in an ocular coloboma phenotype, scale bar 500 *µ*m. (**C**) Histological analysis confirming loss of optic fissure fusion in *ntn1a* morphants. Scale bar 50 *µ*m.

**Figure 8:**
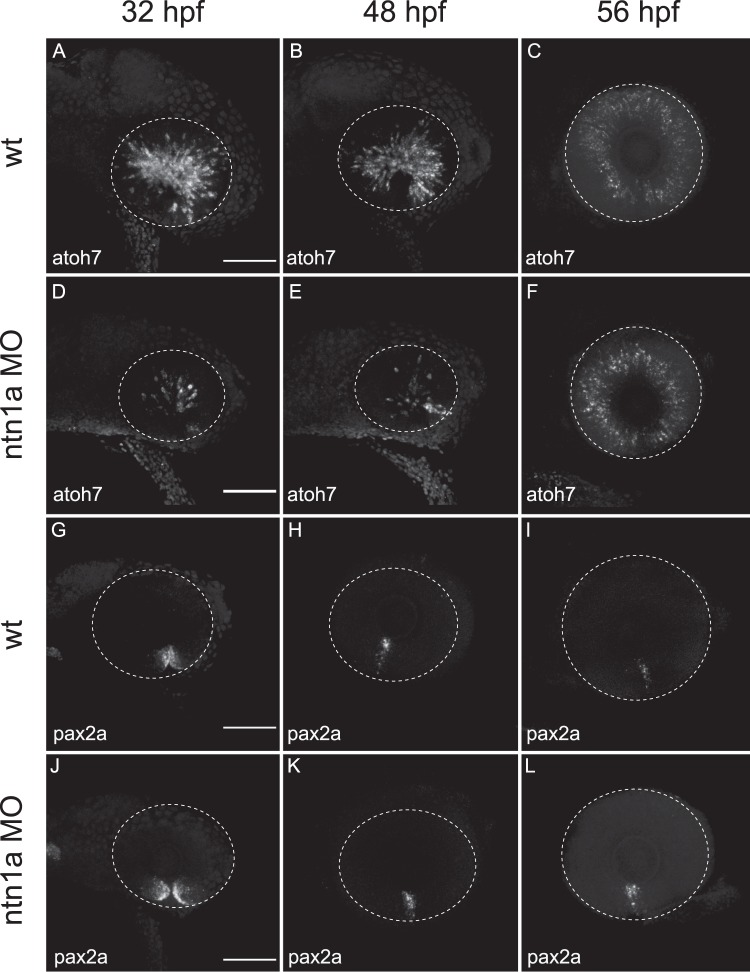
Expression analysis of *ntn1a* morphant embryos through optic fissure fusion. Representative images of *in situ* hybridisation in wild-type and *ntn1a* morphant zebrafish retina using mRNA probes for (**A**–**F**) *atoh7* and (**G**–**L**) *pax2a* at 32, 48 and 56 hpf. White dotted lines indicate the circumference of the eye. Scale bar 50 *µ*m.

To assess the functional role of novel genes having higher expression in OF over time compared to DR, we undertook morpholino antisense translation-blocking knockdown of two candidates*, fam132a* and *ntn1a*, not previously related to fusion clustered in the LRT analysis (Fig. 6). Morphant *fam132a* embryos demonstrated no discernible phenotype on wholemount morphology (data not shown), however the reduction of *ntn1a* resulted in loss of optic fissure fusion at 48 hpf and 56 hpf timepoints (Fig. 7B,C). No significant difference was observed at 32 hpf (Fig. 7C). Temporal expression of *ntn1a* from the RNA-seq data showed significantly higher expression in the optic fissure at 32 hpf, decreasing over 52 hpf (Fig. 7A). *In situ* analysis of specific genes related to coloboma/optic fissure fusion/eye development (*vax1/2, pax2a, eomesa*, and *atoh7*) were carried out on 32, 48 and 56 hpf staged wt and *ntn1a* morphant embryos. The expression pattern of *atoh7*, critical for the develop of retinal ganglion cells and optic nerve formation^33^, was significantly reduced in *ntn1a* morphant embryos at 32 hpf and 48 hpf compared to wt (Fig. 8A,B,D,E). However, at 56 hpf the expression of *atoh7* was similar in wt and *ntn1a* morphants (Fig. 8C,F). Expression of *pax2a* remained persistent in *ntn1a* morphant embryos at 56 hpf compared to wt (Fig. 8I,L). Analysis of the expression of eomesa and vax1/2 showed no difference between morphants and controls, however showed expression patterns validating the RNA-seq data between OF and DR tissues (data not shown).

## Discussion

During zebrafish development, at 32 hpf despite tight opposition of the optic fissure margins, the basement membrane remains intact throughout the proximal-distal axis of the fissure and by 48 hpf, basement membrane breakdown has commenced^5^. Optic fissure fusion is complete by 56 hpf^5,24^. Through analysis of genome-wide expression patterns within tissue dissected from the OF and opposing DR at these three key developmental stages, we have identified DEGs and highlighted functional characteristics of gene-sets during optic fissure fusion. Our analyses captured molecular signatures of ocular development, which could further be exploited to help elucidate the mechanisms that underpin normal and aberrant optic fissure fusion.

Pairwise condition testing revealed the highest number of significant DEG events between OF and DR at 32 hpf, anticipated by stark differences in tissue morphology between the unfused OF and the opposing DR at this time. Notably, many of the genes differentially expressed between OF and DR tissue at 48 hpf, were also differentially expressed at 56 hpf, indicating a level of homogeneity in gene expression in OF once the margins of the fissure have opposed at 48 hpf. Overrepresentation and hierarchical cluster analysis of pairwise condition test data, facilitated the enrichment of known biological functions, interactions and pathways, providing biological insight to the data. For example, optic fissure morphogenesis involves a complex series of dynamic tissue rearrangements at the ventral side of the optic cup and optic stalk; the margins of the fissure grow and fuse to form a seamless conduit through which the POM and hyaloid vasculature enters the eye. This spectrum of cellular events is sensitive to genetic perturbation. A high degree of concordance in gene expression intensities between RNA-seq and qRT-PCR, as indicated by Spearman’s correlation coefficient indicated that, for most genes, our RNA-seq data provides a highly reliable estimate of gene expression levels during optic fissure closure.

Importantly, our RNA-sequencing data provided information on the expression of a number of known or putative ocular patterning, optic fissure fusion and/or ocular coloboma-causing genes. Using time-course analysis to define dynamic changes in global gene expression over time allowed the prioritisation of highly significant DEG events between OF and DR for further functional investigation.

Netrins are secreted proteins that mediate cell migration, cell-cell interactions and cell-extracellular matrix adhesion^34^ and may therefore govern the morphogenetic movements that lead to optic fissure fusion. Zebrafish *ntn1a* expression has been described in the cells that flank the zebrafish optic fissure at 32–36 hpf^35^ and *ntn1a* expression is reduced at the optic fissure of coloboma *pax2a* (*noi*) mutants^36^. During ocular development, *ntn1a* has been shown to mediate retinal ganglion cell axonal pathfinding and angiogenesis at the optic disc^37^ however, its role in optic fissure fusion has not been described. Here, we show that during wild-type ocular development, *ntn1a* expression is lost at the optic fissure margin prior to fusion and that morpholino-mediated depletion of *ntn1a* leads to ocular coloboma, confirming its role as a novel mediator of optic fissure fusion and validating our systematic approach to RNA-sequencing data analysis and candidate gene prioritisation. The corresponding down-regulation of *atoh7*, a modulator of ganglion cell differentiation, confirms the functional loss of *ntn1a*, a known stimulator of axonal outgrowth.

The gene *fam132a*, also known as *c1qtnf12* (C1q and TNF related 12, CTRP12) has no previous expression data contained in the ZFIN database. As a significant DEG event we chose *fam132a* for translation-blocking morpholino knockdown. No altered phenotype was observed at the time points, compared to wild-type zebrafish. The C1QTNF gene family has many members, each encompassing a complement component C1q domain. This is a globular domain which forms a trimer as the basic unit, but also potentially assembles into higher multimeric structures^38,39^. Although no identified paralogues of *fam132a* are present in the GRCz10 genome build, we isolated a sequence termed si:ch1073-184j22.1 through amino acid conservation with the mammalian *fam132b* sequence. Due to the nature of the C1QTNF gene family, functional compensation by redundant genes may prevent a phenotype being observed or this allele may not be fully disruptive to protein function.

Further work is required to elucidate the function of other significant DEG events and their interactors identified via the time-course analysis, specifically, gene clusters showing precise expression across the dorsoventral axis of the developing retina, which are likely relevant during this developmental process. This will contribute to our understanding of unknown cell behaviours central to optic fissure morphogenesis.

## Conclusion

Our study represents the first detailed temporal analysis of the zebrafish optic fissure transcriptome. We have discussed characteristic biological signatures inferred from overrepresentation analysis of DEG events and highlighted *ntn1a* as a novel mediator required for optic fissure fusion. This data not only informs the molecular basis of optic fissure fusion but will contribute to the validation of genetic variants discovered in human patients, the elucidation of pathological pathways, and the development of novel therapeutics for ocular coloboma.

## Materials and Methods (see Supplementary Information for detailed methods)

### Zebrafish husbandry

Zebrafish (wild-type, AB-strain [wt]) were bred and maintained according to local UCL and UK Home Office regulations for the care and use of laboratory animals under the Animals Scientific Procedures Act at the UCL Institute of Ophthalmology animal facility. UCL Animal Welfare and Ethical Review Body approved all procedures for experimental protocols, in addition to the UK Home Office (License no. PPL PC916FDE7). All approved standard protocols followed the guidelines of the ARVO Statement for the Use of Animals in Ophthalmic and Vision Research Ethics^40,41^.

### RNA collection and sequencing

Optic fissure and opposing dorsal retinal tissue was dissected from 5 biological replicates at 32 hpf, 48 hpf and 56 hpf. RNA was extracted using the RNeasy FFPE Kit (Qiagen) and quantified using the Bioanalyzer 2100 RNA Pico system (Agilent biosystems). cDNA libraries were constructed from total RNA (RIN ≥ 8) using the Clontech SMART-Seq v4 Ultra Low Input RNA Kit for Sequencing (Clontech) and sequenced on a HiSeq 2500 system using v4 chemistry (Illumina). Paired-end sequences of 100 bp were generated.

### RNA-seq data analysis

The FASTQ files were assessed for quality control using FASTQC (v0.11.5). Adapter sequences were removed using Trimgalore (v0.4.3). Reads were aligned to the Ensembl *D. rerio* reference genome (build GRCz10). Counts were summarised using HTSeq (v0.6.1) after read duplicates were marked using Picard tools. Differential expression analysis including successive pairwise condition testing and likelihood ratio tests (LRT) was performed using DESeq2 (v1.18.1). DEGs were subjected to GO enrichment analysis using the Bioconductor R package GOseq (v1.32.0), Gorilla^42^ and WebGestalt^43^.

### Quantitative RT-PCR and *in situ* hybridisation

RT-qPCR was performed on the StepOnePlus Real-Time PCR system (Applied Biosystems) using SYBR Select Master Mix (Applied biosystems). mRNA levels were measured in triplicate and normalised to *znf644a* and *g6pd* (Primer sequences are detailed in Table S4). RNAscope *in situ* hybridisation was performed as per the manufacturer’s protocol. Slides were mounted in Prolong Gold Antifade mountant (Life Technologies) and imaged using a Leica LSM 700 confocal microscope.

### Morpholino injections

25-mer morpholinos (MOs; Gene Tools, LLC Philomath, Oregon) were designed to target the ATG translation start site for both the *ntn1a* and *fam132a* mRNA transcripts. One cell stage embryos were co-injected with 10 nl of 5 pg of GFP mRNA and 2.5 pmol of *ntn1a, fam132a* or control morpholino. Morpholino sequences: *ntn1a* (5′ CAT CAG AGA CTC TCA ACA TCC TCG C 3′), *fam132a* (5′ CAG CTA GTA CCC AGC AAC GCA TCT T 3′), and control (TGT TGA AAT CAG CGT GTT CAA G).

## Supplementary information

Supplementary Information

Supplementary Dataset 2

Supplementary Dataset 3

Supplementary Dataset 4

Supplementary Dataset 5

## Data Availability

All data generated or analysed during this study are included in this published article (and its Supplementary Information files).
