# Supplementary material for: Transcriptome profiling of zebrafish optic fissure fusion
Source: Sci Rep. 2019 Feb 7;9:1541. doi: 10.1038/s41598-018-38379-5 (PMC6367446; doi:10.1038/s41598-018-38379-5)
Supplement: Supplementary file 1 — Supplementary Information [file 41598_2018_38379_MOESM1_ESM.pdf]

Title: Transcriptome profiling of zebrafish optic fissure fusion

Author affiliation: R Richardson[1], N Owen[1], M Toms[1], R Young[1], D Tracey-White[1], M Moosajee[1,2,3]

1 - Development, Ageing and Disease, UCL Institute of Ophthalmology, London, UK

2 - Department of Genetics, Moorfields Eye Hospital NHS Foundation Trust, London, UK

3 - Department of Ophthalmology, Great Ormond Street Hospital for Children NHS Foundation Trust, London, UK

Supplementary Information

Detailed materials and methods

RNA collection and sequencing

Eye diameters at 32, 48 and 56 hpf were estimated by imaging fifty staged embryos from a single clutch. Horizontal eye diameter was measured in the developing eye using ImageJ. Ten such clutches per timepoint were collected and measured independently and the population means of the eye diameters, estimated from the means of clutch replicates, were determined to be 186.1 µm for 32 hpf (95% CI = 178.0 µm—194.1 µm), 212.7 µm for 48 hpf (95% CI = 206.7 µm—218.6 µm) and 232.4 µm for 56 hpf (95% CI = 223.5 µm—241.3 µm). During sample dissection, eye diameter was measured and only those that fell within the 95% confidence intervals of the population mean, and fulfilled conventional staging criteria, were chosen for dissection. Staged embryos were fixed for 1-2 hours by immersion in RNase-free 4% paraformaldehyde at 4°C. Optic fissure (OF; ~50 µm from either side of the fissure) and opposing dorsal retinal tissue (DR; ~100 µm) was dissected using fine tip tweezers and a sapphire blade (World precision instruments), snap-frozen and stored for RNA extraction. Tissue was dissected from 5 biological replicates per timepoint; each biological replicate was generated from an independent breeding pair. RNA was extracted using the RNeasy FFPE Kit (Qiagen) and quantified using the Bioanalyzer 2100 RNA Pico system (Agilent biosystems) per manufacturer's instructions. Samples of RIN ≥ 8 were selected for sequencing. cDNA libraries were constructed from total RNA using the Clontech SMART-Seq v4 Ultra Low Input RNA Kit for Sequencing (Clontech) and sequenced on a HiSeq 2500 system using v4 chemistry (Illumina). Libraries were paired-end sequenced to a depth of between 20-40 million reads at a length of 100 bp. Library preparation and sequencing were performed by Eurofins Genomics (Ebersberg, Germany).

RNA-seq data analysis

Raw sequencing reads per sample per lane were concatenated and read quality assessed. Initially samples were evaluated with FASTQC (v0.11.5) demonstrating between 40-80% duplication of reads per sample. Samples with extreme duplication rates were removed from the analysis. Adapter and low quality scored base reads (Phred < 6) were removed using Trimgalore (v0.4.3). High-quality cleaned FASTQ reads were aligned to the Ensembl *D.rerio* reference genome (build GRCz10) and annotation (build GRCz10.89) downloaded June 2017 using the splice-aware aligner STAR (v2.5.2b). Resultant BAM files were sorted, indexed, duplicates marked with Picard tools and subsequently used to generate count files using HTSeq (v0.61,[1]). To assess mapping efficiencies, aligned reads were analysed with RNA-SeQC (v1.1.8,[2]). Statistics of RNA-seq reads and mapping were outlined in supplementary file SI_01. Duplicate reads were not removed from the count data, however *D.rerio* rRNA and

tRNA species were. Count files generated using HTSeq were processed for subsequent differential expression analysis with the Bioconductor R packages tximport [3] and DESeq2 (v1.18.1,[4]). PCA performed on the regularised log transformed count data in DESeq2.

To investigate differential gene expression we performed successive pairwise condition testing (i.e. OF tissue 32 hpf vs OF tissue 48 hpf, OF tissue 48 hpf vs OF tissue 56 hpf, and so on) using the raw count data. DESeq2 was used to model the counts to the negative binomial GLM, normalising to library size (factor), and identify differentially expressed gene (DEG) events using a combinational factor of tissue (OF/DR) and time (32/48/56). Resulting *p* values were adjusted for multiple testing, as part of the DESeq2 analysis, using Benjamini-Hochberg correction[5]. Classification of a differentially expressed gene was set according to the following criteria; adjusted *p* value of ≤ 0.01 and fold change cutoff of two-fold change between conditions. To further investigate changes over time to obtain a complete picture of expression in the dataset we carried out analysis with DESeq2 LRT (likelihood ratio tests) analysis. To assess the interaction of time with the tissue origin on gene expression we tested the full design matrix formula of ~ tissue + time + tissue:time and reduced matrix design formula ~ tissue + time. The reference levels in the DESeq2 object were set as the DR tissue and time 32 hpf.

Visualisation of DEG events was carried out using the Bioconductor package NMF[6]. Significant DE Ensembl gene ID lists were generated and heatmaps produced through clustering using hierarchical average linkage clustering and Euclidean distances.

Gene Ontology Analysis

Gene Ontology (GO) analysis of DEGs was performed using a combination of Bioconductor packages; GOseq[7], GenomicFeatures[8] and Pathview[9]. GOseq was used with correction for gene length bias in the RNA-seq data. The GO hierarchy “Biological process” was used to query these genes against the background of all genes expressed the RNA-seq dataset. Only GO terms with an adjusted $p < 0.05$ were considered. Redundant GO terms were removed with REViGO [10].

Quantitative real-time PCR analysis (qRT-PCR)

For verification of transcript changes identified in RNA-seq, OF and opposing DR tissue was dissected from ten 32, 48 and 56 hpf embryos (one eye per embryo) and pooled for RNA extraction. Each pool was defined as one of three biological replicates, obtained from independent breeding pairs. RNA was extracted using the RNeasy FFPE Kit (Qiagen), quantified using the RNA Analysis 2200 ScreenTape system (Agilent Genomics) and

converted to cDNA using the Superscript III First-Strand Synthesis SuperMix for qRT-PCR Kit (Invitrogen) per manufacturer's instructions. All qRT-PCR was performed on the StepOnePlus Real-Time PCR system (Applied Biosystems) using SYBR Select Master Mix (Applied biosystems). Primer sequences are listed in supplementary file SI_04. mRNA levels were measured in triplicate for each biological replicate and normalised to reference genes *g6pd* and *znf644a*, chosen for their stable expression across samples. Genes were selected using calculated transcripts per million (TPM) values for each sample that showed the lowest variation between samples and were previously reported as reference genes. Correlation in expression intensities between RNA-seq and qRT-PCR was calculated using the Spearman correlation coefficient.

RNAscope RNA *in situ* hybridization

Zebrafish embryos were fixed by immersion in 4% paraformaldehyde/PBS at 4°C overnight. After washing in PBS, embryos were cryo-protected by successive 10%, 20% and 30% sucrose/PBS washes at 4°C before cryo-embedding in Tissue-Tek O.C.T embedding medium (VWR). 12 µm sections were collected onto Superfrost PLUS slides (VWR). Briefly, tissue was washed with PBS for 5 minutes, boiled in RNAscope Target Retrieval reagent (Advanced Cell Diagnostics, ACD) for 5 minutes, washed with sterile water and incubated in RNAscope Protease III reagent (ACD) for 15 minutes at 40°C. Tissue was hybridised with *vax1*, *bambia*, *pax2a*, *tbx3a* and *rbp2a* target probes and negative control probe *dapB* for 2 hours at 40°C. Probes were designed by ACD. Hybridization signals were amplified and visualized with the RNAscope Fluorescent Multiplex Detection kit (ACD) per manufacturer's instructions. Tissue was washed in PBS immediately after *in situ* hybridisation of RNA targets and slides were mounted in Prolong Gold Antifade mountant (Life Technologies) and imaged using a Leica LSM 700 confocal microscope.

Wholemount i*n situ* hybridisation

Whole mount in situ hybridisation was performed using digoxigenin (DIG)-labelled RNA probes according to standard protocols[11]. Probes were synthesized using T7 or T3 RNA polymerases (Promega) according to manufacturers' instructions and supplied with DIG labelled UTP (Roche). Probes were detected with anti-DIG-POD (1:5000, Roche) antibodies and developed with Fluorescein tyramide substrate for confocal analysis[12].

Morpholino injections

25-mer morpholinos (MOs; Gene Tools, LLC Philomath, Oregon) were designed to target the ATG translation start site for both the *ntn1a* and *fam132a* mRNA transcripts. A 5-mispair MO derived from each ATG targeted MO was used as a control. Each MO sequence was BLAST

searched on the NCBI database against the zebrafish genome to ensure that there would not be any non-specific mRNA/morpholino hybridization. One cell stage embryos were co-injected with 10nl of 5pg of GFP mRNA and 2.5 pmol of *ntn1a*, *fam132a* or control morpholino. Only embryos with an even distribution of GFP fluorescence were used for experiments. Morpholino sequences: *ntn1a* (5' CAT CAG AGA CTC TCA ACA TCC TCG C 3'), *fam132a* (5' CAG CTA GTA CCC AGC AAC GCA TCT T 3'), and control (TGT TGA AAT CAG CGT GTT CAA G). Co-injection of p53 morpholino was carried out with the gene-specific morpholinos as control experiments.

References

1 Anders, S., Pyl, P. T. & Huber, W. HTSeq--a Python framework to work with high-throughput sequencing data. *Bioinformatics* **31**, 166-169, doi:10.1093/bioinformatics/btu638 (2015).

2 DeLuca, D. S. *et al.* RNA-SeQC: RNA-seq metrics for quality control and process optimization. *Bioinformatics* **28**, 1530-1532, doi:10.1093/bioinformatics/bts196 (2012).

3 MD, S. C. L. M. R. Differential analyses for RNA-seq: transcript-level estimates improve gene-level inferences. *F1000Research* **4** (2015).

4 Love, M. I., Huber, W. & Anders, S. Moderated estimation of fold change and dispersion for RNA-seq data with DESeq2. *Genome Biol* **15**, 550, doi:10.1186/s13059-014-0550-8 (2014).

5 Benjamini, Y. & Hochberg, Y. Controlling the False Discovery Rate - a Practical and Powerful Approach to Multiple Testing. *Journal of the Royal Statistical Society Series B-Methodological* **57**, 289-300 (1995).

6 Gaujoux, R. & Seoighe, C. A flexible R package for nonnegative matrix factorization. *BMC Bioinformatics* **11**, 367, doi:10.1186/1471-2105-11-367 (2010).

7 Young, M. D., Wakefield, M. J., Smyth, G. K. & Oshlack, A. Gene ontology analysis for RNA-seq: accounting for selection bias. *Genome Biol* **11**, R14, doi:10.1186/gb-2010-11-2-r14 (2010).

8 Lawrence, M. *et al.* Software for computing and annotating genomic ranges. *PLoS Comput Biol* **9**, e1003118, doi:10.1371/journal.pcbi.1003118 (2013).

9 Luo, W. & Brouwer, C. Pathview: an R/Bioconductor package for pathway-based data integration and visualization. *Bioinformatics* **29**, 1830-1831, doi:10.1093/bioinformatics/btt285 (2013).

10 Supek, F., Bosnjak, M., Skunca, N. & Smuc, T. REVIGO summarizes and visualizes long lists of gene ontology terms. *PLoS One* **6**, e21800, doi:10.1371/journal.pone.0021800 (2011).

11 Thisse, C. & Thisse, B. High-resolution in situ hybridization to whole-mount zebrafish embryos. *Nat Protoc* **3**, 59-69, doi:10.1038/nprot.2007.514 (2008).

12 Lauter, G., Soll, I. & Hauptmann, G. Multicolor fluorescent in situ hybridization to define abutting and overlapping gene expression in the embryonic zebrafish brain. *Neural Dev* **6**, 10, doi:10.1186/1749-8104-6-10 (2011).

SI_01_RNA-seq dataset alignment metrics

Sample	Total Reads Sequenced	Mapped	Mapped Unique	Exonic Rate	Intragenic Rate	Over Exons Rate	Intronic Rate	Genes Detected
1-32-O	33924614	27515575	5676246	80.06%	7.82%	3.61%	12.12%	13270
1-32-R	15721920	13077795	2906490	82.13%	7.08%	3.62%	10.79%	11222
1-48-O	25520054	24433117	17776436	76.28%	8.17%	3.51%	15.55%	18275
1-48-R	23426120	22420598	16892001	77.65%	7.77%	3.45%	14.57%	17881
1-56-O	26106984	24854484	17158426	73.17%	10.01%	3.48%	16.83%	19502
1-56-R	26571602	24788399	17884464	50.75%	20.35%	3.49%	28.89%	21854
2-32-O	26937960	25779516	18532230	81.81%	5.99%	3.63%	12.20%	16342
2-32-R	25470616	24486415	18240904	83.50%	5.38%	3.47%	11.13%	16402
2-48-O	26065822	24996295	17713464	76.27%	8.35%	3.43%	15.38%	18992
2-48-R	30921406	29705251	20254128	80.64%	6.75%	3.34%	12.61%	18359
2-56-O	26763344	25604291	19639877	75.26%	8.85%	3.38%	15.88%	19378
2-56-R	27365670	26191780	19096793	77.82%	7.83%	3.50%	14.35%	18462
3-32-O	27375456	26338640	19344530	81.64%	5.66%	3.47%	12.70%	17508
3-32-R	22464670	21624259	16374677	82.87%	5.36%	3.41%	11.77%	16363
3-48-O	36179364	34638249	23162259	76.95%	8.10%	3.52%	14.95%	19089
3-48-R	30155606	28738736	19862638	77.35%	8.18%	3.61%	14.47%	17505
3-56-O	29622222	28312719	19941256	77.83%	8.23%	3.37%	13.94%	18647
3-56-R	28366558	27112000	18820668	79.55%	7.34%	3.45%	13.11%	18383
4-32-O	28621468	27479778	20509597	84.21%	5.44%	3.33%	10.35%	16632
4-32-R	25685948	24561425	18842873	83.33%	5.50%	3.41%	11.17%	15942
4-48-O	29727870	28496693	20126051	79.33%	7.27%	3.34%	13.41%	18500
4-48-R	28446712	27255378	18519068	80.46%	6.77%	3.37%	12.77%	17646
4-56-O	25753550	28179375	19429790	77.12%	8.58%	3.26%	14.30%	18446
4-56-R	25753550	24653749	13263226	80.83%	6.87%	3.35%	12.31%	17720
5-32-O	28122930	27010338	19799028	84.00%	4.98%	3.32%	11.02%	16853
5-32-R	30646558	29415509	21281825	84.39%	5.01%	3.36%	10.59%	16061
5-48-O	28336056	27216863	19191815	80.56%	6.66%	3.32%	12.78%	18655
5-48-R	22399212	21558549	15491177	80.14%	6.60%	3.36%	13.26%	18158
5-56-O	29758786	28560435	17307866	77.51%	7.97%	3.23%	14.51%	18469
5-56-R	30216782	28976313	15909253	80.38%	6.85%	3.22%	12.76%	17812
